# Matrix-Assisted Laser Desorption/Ionization Time-of-Flight Mass-Spectrometry (MALDI-TOF MS) Based Microbial Identifications: Challenges and Scopes for Microbial Ecologists

**DOI:** 10.3389/fmicb.2016.01359

**Published:** 2016-08-30

**Authors:** Praveen Rahi, Om Prakash, Yogesh S. Shouche

**Affiliations:** Microbial Culture Collection, National Centre for Cell SciencePune, India

**Keywords:** MALDI-TOF mass spectrometry, microbial identification, spectral database, culturomics, high-throughput identifications

## Abstract

Matrix-assisted laser desorption/ionization time-of-flight mass-spectrometry (MALDI-TOF MS) based biotyping is an emerging technique for high-throughput and rapid microbial identification. Due to its relatively higher accuracy, comprehensive database of clinically important microorganisms and low-cost compared to other microbial identification methods, MALDI-TOF MS has started replacing existing practices prevalent in clinical diagnosis. However, applicability of MALDI-TOF MS in the area of microbial ecology research is still limited mainly due to the lack of data on non-clinical microorganisms. Intense research activities on cultivation of microbial diversity by conventional as well as by innovative and high-throughput methods has substantially increased the number of microbial species known today. This important area of research is in urgent need of rapid and reliable method(s) for characterization and de-replication of microorganisms from various ecosystems. MALDI-TOF MS based characterization, in our opinion, appears to be the most suitable technique for such studies. Reliability of MALDI-TOF MS based identification method depends mainly on accuracy and width of reference databases, which need continuous expansion and improvement. In this review, we propose a common strategy to generate MALDI-TOF MS spectral database and advocated its sharing, and also discuss the role of MALDI-TOF MS based high-throughput microbial identification in microbial ecology studies.

## Introduction

Microorganisms are ubiquitous in all ecosystems, are intimately associated with environmental sustainability and human development, and perform various ecological functions. Metagenomics and other cultivation-independent studies showed that a major proportion of the world's microbial communities are not yet cultivated and their functions are unknown. Exploiting the information gathered from metagenomics studies, new cultivation techniques are being designed to achieve cultivation of the enormous diversity of the microorganisms present in an ecosystem, including dominant and rare species (Vartoukian et al., 2010; Stewart, 2012; Dubourg et al., 2014). Recently a strategy to understand the functional and ecological capacities of microorganisms has been proposed, which include a combination of functional phylogenomics and co-culture-based experiments (Cibrián-Jaramillo and Barona-Gómez, 2016). To meet this goal, high-throughput cultivation techniques are being used, which yield large numbers of microbial isolates. Rapid and accurate characterization of such large number of microorganisms is always a big challenge for any microbiologist. Traditionally, various morphological, physiological and biochemical features were employed for microbial identifications. Application of molecular tools like, small subunit ribosomal RNA gene sequencing, whole genome sequencing, multi-locus sequence typing (MLST), amplified fragment length polymorphism (AFLP), and DNA-DNA hybridization (DDH) enhanced the resolution of microbial identification (Müller et al., 2007; Ragimbeau et al., 2008). At present the polyphasic approach provides the most reliable identification of microorganisms but it is time consuming, expensive, and requires expertise in both conventional as well as in modern microbiological techniques. When dealing with small number of microorganisms, speed, and cost of characterization may not be of considerable importance but are very critical when a larger number of microorganisms are being analyzed.

MALDI-TOF MS has gained popularity as a microbial biotyping tool due to its speed, low-cost, simplicity, and applicability for a wide range of microbes including bacteria, archaea, and fungi especially in clinical set-ups. It is also becoming an increasingly essential technique for microbial characterization and identification in environment microbiology and microbial diversity studies. The ability of this technique to differentiate at the species level has been found useful in describing new species (Koziel et al., 2014; Lang et al., 2015; Patil et al., 2015; Tong et al., 2015). Realizing the importance of MALDI-TOF MS in microbial taxonomy, identification and diversity analysis, the journal Systematic and Applied Microbiology published a special issue on this subject in 2011 (vol. 34). Concept, chemistry, loopholes, and application of the technique have already been discussed earlier by several authors (Seng et al., 2009; Moore and Rosselló-Móra, 2011; Steensels et al., 2011; Welker and Moore, 2011; Murray, 2012; Wieser et al., 2012; Fournier, 2013). However, none of these reviews deals directly with extension and development of MALDI spectral database, and the application of MALDI-TOF MS to microbial ecology. In brief, it requires mixing of microbial cells from a pure colony with the matrix solution, followed by smear preparation on target plate, air drying, loading of plate inside instrument and finally exposing the samples to source of ionization. After ionization, ionized peptides, and proteins travel toward detector in a vacuum tube, and get separated based on their mass to charge ratio (m/z). Finally, mass spectrum of the strain under study is compared with that of the other strains present in the reference database. The database includes biomarkers spectra of intracellular proteins primarily in the range of 2–20 kDa (Fenselau and Demirev, 2001; Benagli et al., 2012). Most of the biomarkers detected in MALDI-TOF spectra of intact bacterial cells have a molecular mass below 15 kDa (Ryzhov and Fenselau, 2001; Croxatto et al., 2012; Suarez et al., 2013). Lysis of organisms with organic solvents in acidic conditions favors extraction of basic cytoplasmic proteins. Multiple commercial platforms including Bruker-Biotyper (Bruker Daltonics, Bremen, Germany), Axima Assurance (Shimadzu, Kyoto, Japan), SARAMIS AnagnosTec (AnagnosTec GmbH, Potsdam, Germany), and Andromas (Andromas SAS, Paris, France) have been developed for microbial identification (Risch et al., 2010; Marko et al., 2012). SARAMIS AnagnosTec was acquired by bioMérieux and redeveloped with the name of Vitek-MS (bioMérieux, Marcy L'Etoile, France; Patel, 2013).

In this review, we discuss the importance of reliable database(s) in the MALDI-TOF MS applications in microbial identification, possibilities to improve and expand the limited database(s) with special emphasis on samples of non-clinical origin. The review also provides brief information about its potential applications in microbial ecological studies.

## Accuracy and resolution of MALDI-TOF MS in microbial identification

MALDI-TOF MS is a rapid and reliable technique for microbial identification as most results obtained using this are similar to that of 16S rRNA gene sequence analysis but at a rapid rate and at a lower cost. This technique is based on fingerprinting analyses of primarily ribosomal proteins, which are synthesized under all growth conditions and are the most abundant cellular proteins (Ryzhov and Fenselau, 2001). Many reports indicated that the technique generated more accurate identification results than the ones based on various phenotypic and biochemical tests conventionally used in microbiological laboratories (Seng et al., 2009; van Veen et al., 2010; Bessède et al., 2011; Bizzini et al., 2011; Carbonnelle et al., 2012). Resolution of certain taxonomic groups, like *Bacillus cereus* complex, *Brukholderia cepacia* complex, *Escherichia coli* and *Shigella* group, *Enterobacter cloacae* complex, and *Pseudomonas putida* complex, still remain a daunting challenge for routine MALDI-TOF MS analysis and 16S rRNA gene sequencing (Pavlovic et al., 2012; Khot and Fisher, 2013; Almuzara et al., 2015). Improved analysis software and new approaches are being used to further increase the taxonomic resolution of MALDI-TOF MS based biotyping, especially for members of such groups and complexes (Fernández-No et al., 2013; Khot and Fisher, 2013; AlMasoud et al., 2014). For example, application of ClinPro Tools software (Bruker Daltonics) was very useful in rapid differentiation of closely related *Shigella* species from *E*. *coli* species, which could not have been achieved by methods like 16S rRNA gene sequencing and routine MALDI-TOF MS analysis (Khot and Fisher, 2013). Advantages and limitations of MALDI-TOF MS based microbial characterization to achieve higher taxonomic resolution at different levels have been discussed in details in a recent review (Sandrin et al., 2013).

## Role of databases in MALDI-TOF MS based identifications

Like other automated microbial identification systems (MIDI, Vitek, Biolog etc.) MALDI-TOF MS also relies on a reference database for identification of microorganisms. Although instruments made by different manufacturers follow similar principle, the major differences remains in the procedure and algorithm used in creating their own reference databases (Welker, 2011; Rychert et al., 2013). For example, multiple spectra of a strain were used to generate strain specific “main spectra profile (MSP)” in the Biotyper (Bruker Daltronics) database (Sauer et al., 2008). Vitek MS (BioMerieux), on the other hand, generate a single “Super Spectrum” from spectra of 10 strains belonging to one species and growing them on different media and incubation conditions (Emonet et al., 2010). Differences in approaches to create separate databases are reflected in the level of accuracy in identification of the same set of microorganisms as presented in Table 1.

**Table 1:** **Comparative studies on identification of microorganisms using different MALDI-TOF MS platforms and databases from the time point of the published work**.

**Organism (nos.)**	**MALDI-TOF MS system and database**	**Results**	**References**
Bacteria from clinical sources (720)	Bruker Biotyper	High confidence identifications (94%) and incorrect identification (0.9%).	Cherkaoui et al., 2010
	Shimadzu MS with Saramis database	High confidence identifications (89%) and incorrect identification (0.5%).	
Bacteria isolated in routine at hospital (317)	Bruker Biotyper (ver. 2.0)	Correct identification to genus level (97.4%) and species level (94.9%).	Carbonnelle et al., 2012
	Axima Assurance system Shimadzu/SARAMIS (Anagnos Tec GmbH ver. 2008)	Correct identification to genus level (97.2%) and species level (93.4%).	
Selected bacterial strains with low discrimination with phenotypic tests, unusual antimicrobial susceptibility and fastidious cultures (296)	Bruker Biotyper (ver. 2.0)	Correct identification to genus level (94.9%) and species level (83.4%).	Carbonnelle et al., 2012
	Axima Assurance system Shimadzu/SARAMIS (Anagnos Tec GmbH ver. 2008)	Correct identification to genus level (83.8%) and species level (65.9%).	
Non-fermenting Gram-negative bacilli isolated from cystic fibrosis (200)	Bruker Biotyper (ver. 3.0)	Overall identification (97%), including species level (72.5%), complex level (5.5%) and genus (19.0%), and no identification (3.0%).	Marko et al., 2012
	Vitek MS (SARAMIS ver. 3.62)	Overall identification (93%), including species level (80.0%), complex level (3.5%), genus (6.0%) and family (3.5), and no identification (7.0%).	
Bacteria from multiple clinical sources (986)	Bruker Biotyper (ver. 3.0)	Genus and species level (92.7%), genus level (2.8%) and no identification (3.2%). Misidentification to species level (1.2%) and genus level (0%).	Martiny et al., 2012
	Vitek MS (IVD v1)	Genus and species level (93.2%), genus level (0.4%) and no identification (5.8%). Misidentification to species level (0.4%) and genus level (0.2%).	
	Vitek MS (Saramis database)	Genus and species level (83.8%), genus level (3.1%) and no identification (12.8%). Misidentification to species level (0.3%) and genus level (0%).	
Yeasts isolated from routine clinical specimens (312)	Bruker Biflex III-Biotyper (ver. 2.0.4.0)	Valid identification (87.2%), correct species identification of < 1.7 (5.8%), no identification (6.4%) and false species identification (0.6%).	Lohmann et al., 2013
	Axima-SARAMIS (AnagnosTec ver. 4.07)	Valid identification (82.7%), correct species identification of < 40% (2.9%), no identification (13.1%) and false species identification (1.3%).	

Until recently, manufacturers of MALDI-TOF MS microbial identification systems targeted mostly clinically important microorganisms, thereby limiting its applications in other areas of microbiological research. Limited uses of MALDI-TOF MS based identifications in microbial ecology study have been attributed mainly to lack of reference spectra in the databases associated with the instruments, or inability of the spectra to differentiate very similar species (Bizzini et al., 2011; Seng et al., 2013). Therefore, there is a need to augment the existing MALDI-TOF MS databases with spectra of more diverse microorganisms to increase wider application of this technology. Creation of in-house database supplementing the limited commercial database has been proven to be highly advantageous for the identification of various microorganisms of clinical and non-clinical origin like *Borrelia, Brachyspira, Bradyrhizobium, Leptospira, Nocardia*, and *Rhizobiaceae* (Table 2), which are under-represented in the commercially available databases. It is evident that efforts to create in-house databases are restricted to very specific group of organisms reflecting interest of the laboratories. A boost in the rate of identification is also expected with the inclusion of rare species and recently described species to the database (Lohmann et al., 2013).

**Table 2:** **Expansion and creation of reference spectral database to improve MALDI-TOF MS based identification**.

**Taxonomic group**	**Strains (nos.)**	**Growth conditions**	Replicates strain^−1^ (nos.)^*^	Spectra MSP^−1^ (nos.)^**^	**References**
*Campylobacter*	09	Columbia agar containing 5% sheep blood; microaerobic conditions; incubation time 48 h; temperature 42°C under.	8 (32)	30	Alispahic et al., 2009
*Arcobacter*	05		8 (32)	30	
*Helicobacter*	03		8 (32)	30	
Yeast	109	SDA medium; incubation time 2–3 d; temperature 30°C.	−	−	Stevenson et al., 2010
*Rhizobiaceae*	56	YMA and TY media; incubation time 24 h; temperature 28°C.	12 (36)	20	Ferreira et al., 2011
*Prevotella*	30	Fastidious anaerobic agar with 5% horse blood; anaerobic conditions; incubation time 24–48 h; temperature 35°C.	10 (30)	20	Wybo et al., 2012
*Brachyspira*	09	BAM and TSA media; anaerobic conditions; incubation time 48–72 h; temperature 28°C.	20 (20)	12	Calderaro et al., 2013
Molds	294	SDA medium; incubation time 5 d; temperature 28°C.	−	10	Lau et al., 2013
*Bradyrhizobium*	18	YMA and TY media; incubation time 24, 48, and 72 h; temperature 28°C.	12 (36)	20	Sánchez-Juanes et al., 2013
*Borrelia*	04	BSK-II medium with 6% rabbit serum; incubation time 7 d; temperature 30°C.	10 (40)	25	Calderaro et al., 2014a
*Leptospira*	20	EMJH medium; incubation time 7 d; temperature 30°C.	10 (40)	25	Calderaro et al., 2014b
Yeast	156	SDA medium; incubation time 24–48 h; temperature 35°C.	−	10	Carolis et al., 2014
*Staphylococcus pseudintermedius*	40	Muller Hinton agar with 5% sheep blood.	9 (27)	27	Murugaiyan et al., 2014
*Staphylococcus intermedius*	05				
*Staphylococcus delphini*	12				
*Brachyspira*	30	TSA containing 10% bovine blood; anaerobic conditions; incubation time 2–6 d; temperature 42°C.	12 (36)	20	Warneke et al., 2014
Haloarchaea	32	NHA medium.			
Halophilic methanoarchaea	13	Defined media with NaCl and trimethylamine.	24	10–24	Shih et al., 2015
Halotolerant and non-halophilic methanoarchaea	24	MB/W medium with different methanogensis substrates.			

* *Replicates per samples spotted on MALDI-TOF MS plate, values in parenthesis are number of spectra generated*.

** *Minimum number of spectra used to create one MSP (main spectra profile)*.

## Development of MALDI-TOF MS spectral database

**Figure 1:**
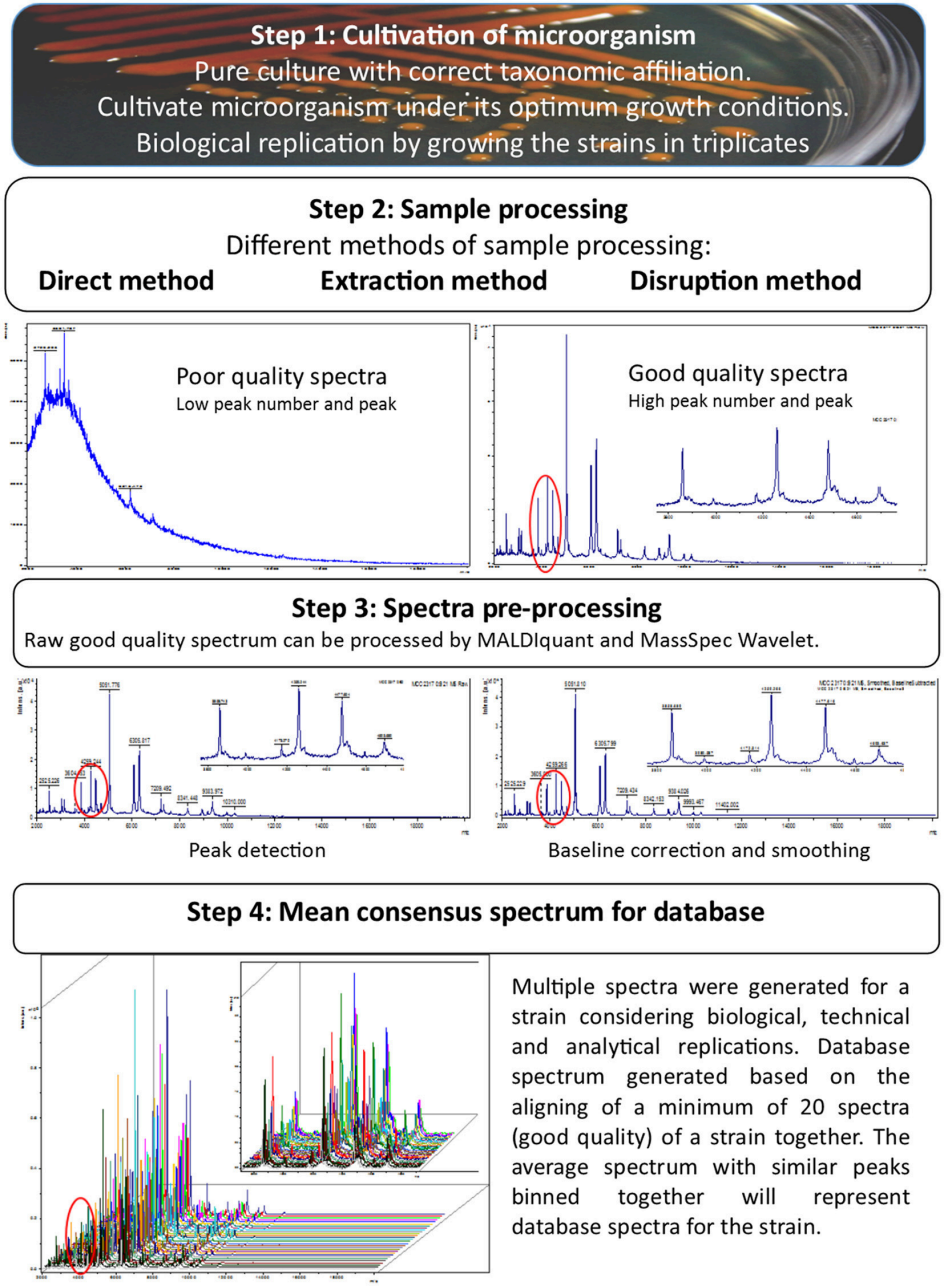
**Stepwise strategy to develop highly stringent and authentic database for MALDI-TOF MS based microbial identification**.

Creating open-source database(s) incorporating reference MALDI-TOF MS spectra of all known species and keep it updated by including newly reported species of microorganisms is a huge task, which requires urgent attention and cooperation of people engaged in this research area. Several researchers have made efforts to expand MALDI-TOF MS spectral database, most of which were done in isolation by creating in-house database. A survey of such independent efforts on in-house database creation revealed number of differences in the procedures of database creation (Table 2). These differences involve mainly growth conditions of microorganisms, replications and minimum number of spectra used to create the mean database spectrum. To develop a universal database the methods for creation and expansion need to be highly standardized. Steps necessary for creating a high quality spectra database are schematically presented in Figure 1. Cultivation of microorganisms as pure culture is prerequisite for MALDI-TOF MS based identification as the contaminated or mixed culture can lead to ambiguous and confusing results. However, researchers used MALDI-TOF MS-based methods to identify complex model mixture containing multiple bacterial strains by using a biomarker-based strategy (Mahé et al., 2014; Zhang et al., 2015). MALDI-TOF MS based characterization of such mixtures directly from environments and of totally unknown microorganisms, where the variability will be inherently higher, has not been optimized so far. Assigning correct taxonomic designation to the strain is highly important before considering it for MALDI-TOF MS spectral database. This can be achieve either by 16S rRNA gene sequencing and/or by using polyphasic approach (including conventional phenotypic and biochemical, and molecular phylogeny).

It has been reported that small variations in growth conditions (i.e., medium composition, pH and temperature) has practically no or very little impact in MALDI-TOF MS based identification of microbes, which contributes toward higher reproducible results in inter-laboratory comparisons (Valentine et al., 2005). Therefore, in order to create good spectral databases it is preferable to use the growth conditions, which can generate sufficient biomass for analysis. Growth phase may also play a role in protein spectra as with longer incubation microorganisms form resistant structures, but this can be avoided by collecting the biomass during actively growing phase. In certain microorganisms, pigments like melanin, carotenoids, and other photoactive compounds might hinder the quality of the spectra obtained. Buskirk et al. (2011) observed the suppression of peptide and protein ion signals during the acquisition of MALDI-TOF mass spectra in the presence of fungal melanin in MALDI sample deposits. Manipulation of culture conditions to block or reduce pigment synthesis can help in generating acceptable MALDI-TOF fingerprint mass spectra from darkly pigmented microorganisms. Important factor that influences the MALDI-TOF MS spectra is sample-processing methods (Freiwald and Sauer, 2009; Alatoom et al., 2011; Cassagne et al., 2011; Matsuda et al., 2012; Hamprecht et al., 2014; Panda et al., 2015). In a comparative study, yeast identification results obtained by using the Vitek MS (with Biomerieux database) and the Bruker Biotyper exhibited that the former platform was more successful in identification, and this difference was explained by fact that same sample processing method (on-plate 25% formic acid extraction) was used for characterization and to generate the Vitek MS Saramis, Biomerieux spectra database (Hamprecht et al., 2014). In contrast, the Bruker Biotyper database was created by ethanol-formic acid extraction. Hence, it is vital to define standard sample processing method(s), which can be used with ease for most of the organisms to generate a high quality spectrum for both proposes (identification and database creation). It is also understood that a single sample processing method could not be applied for all types of microorganisms but can be possible for closely related strains or members of same genus. A clear genus trend was observed for *Actinomyces, Gemella, Nocardia*, and *Streptomyces* species, for which MALDI-TOF MS failed to obtain a sufficient protein signals to generate a quality spectrum by formic acid-acetonitrile extraction (Bizzini et al., 2011). Stafsnes et al. (2013) also reported that few isolates out of 400 isolates did not produce good quality MALDI-TOF MS spectra following extraction with acetonitrile/formic acid/water (50:35:15, v/v) mixture and suggested that the method and solvents used is not sufficient to lyse particular cell wall structures. It is recommended to employ more efficient sample processing methods to get sufficient protein signals. Currently different methods of sample processing, including direct cell, on plate extraction, ethanol-formic acid extraction, trifluoroacetic acid extraction and bead beating extraction are being used at present to acquire good quality MALDI-TOF MS spectra (Box 1). We propose that a good quality spectrum for bacteria should have at least 70–80 peaks and for fungi 30–40 peaks with an average peak intensity of 10^3^ arbitrary units or higher. This proposal of accepting lower peak number for fungi is based on the fact that fungal cultures do not liberate proteins as easy as the bacterial cultures does and the MALDI-TOF MS spectra of fungal cultures generally consist of lesser peaks in comparison to bacterial cultures.

Box 1 **Sample processing methods for MALDI-TOF MS based microbial identification.**

**1. Direct method:**

Direct colony method:

In this method, bacteria were applied, as thin films on the target plates using sterile toothpicks. The bacterial smear were then left to dry at room temperature for 1 min. Subsequently, 1 μl of the matrix solution, comprising a saturated α-cyano-4-hydrocinnamic acid in 50% acetonitrile HPLC grade and 2.5% trifluoroacetic acid, was applied to the samples and co-crystallized with them at room temperature for 10 min (Liu et al., 2007; Freiwald and Sauer, 2009; Matsuda et al., 2012).

**2. Extraction method:**

On plate extraction method:

In this method, each strain was applied and dried on the target plate as in the direct colony method. Following this, 0.5 μl of 70% formic acid was mixed with the sample on the plate by pipetting, followed by 0.5 μl of acetonitrile, and the resultant mixture was dried at room temperature for approximately 10 min. Finally, 1.5 μl of the matrix solution was applied onto the spot as in the direct colony method (Matsuda et al., 2012).

Ethanol-formic acid extraction method:

A loop-full colonies were transferred using a 5 μl inoculating loop into 300 ml of distilled water and 900 ml of ethanol. The suspension was pelleted after centrifugation at 12,000 rpm for 2 min, dried, and then reconstituted in 20–80 ml of 70% formic acid (select volume based on the pellet size). After incubation for 30 s, equal volume of acetonitrile was added. The suspension was then centrifuged at 12,000 rpm for 2 min. A volume of 1.0 μl of the supernatant was applied to spot target plate (Freiwald and Sauer, 2009; Alatoom et al., 2011).

Trifluoroacetic acid extraction method:

Place a loop-full of culture using a 5 μl inoculating loop into a tube and add 50 μl of 80% TFA. Suspend the cell mass by pipetting until complete dissolution and denaturation of the sample achieved. Add 3 volumes of double distilled water (150 μl) and 200 μl of pure acetonitrile and mix by vortexing. Centrifuge at 12,000 rpm at room temperature for 2 min. A volume of 1.0 ml of the supernatant was applied to spot target plate (Freiwald and Sauer, 2009).

**3. Disruption method:**

Physical disruption method:

This procedure is identical to ethanol-formic acid extraction except one additional step that the hydro-alcoholic suspension of sample heated at 95°C for 1 h before extraction. The step of heating can also be replaced with mechanical lyses by 3 cycles of micro-beads beating (60 s) before extraction as described above in ethanol-formic acid extraction (Cassagne et al., 2011).

Enzymatic lysis method:

Microbial biomass were harvested and washed three times with deionized water. The cell pellet resuspended in 30 μl of water were digested by adding 10 μl of 1 mg/ml lysozyme and incubated for 30 min at 37°C. Termination of digestion was accomplished by addition of 0.1% TFA to the pellet (Liu et al., 2007).

We also suggest that the samples should be spotted in triplicates and each spot should be analyzed at least three times generating technical and analytical replicates, to nullify the variations contributed by the technician and the instrument. Ferreira et al. (2011) used 36 independent spectra including biological replicates (two growth media and two incubation temperatures), technical replicates (three spots per sample) and analytical replicates (3 independent measurements for each spot) for each bacterial strain to create reference library of 56 type strains belonging to the family *Rhizobiaceae*. After acquiring multiple good quality MALDI-TOF MS spectra, pre-processing of these newly generated spectra can be done by using software packages available with various MALDI-TOF MS platforms or by using open source software like MALDIquant package and MassSpec Wavelet (Du et al., 2006; Gibb and Strimmer, 2012). After the pre-processing the refined spectra were further aligned to create average spectra and after alignment the peak positions with similar but not identical (i.e., peaks with a variation of ±2 da) were bin together to make all similar peak mass values identical. The resulting final average spectra with similar peaks binned together will represent the database spectra for the test strain.

The in-house databases helped researchers to achieve better identification results (Verroken et al., 2010; Ferreira et al., 2011; Calderaro et al., 2013, 2014a,b; Lau et al., 2013; Sánchez-Juanes et al., 2013; Vávrová et al., 2014; Warneke et al., 2014), but these databases remain inaccessible to other researchers. Development of an open access and universal database incorporating MALDI-TOF MS spectra of as many microorganisms as possible has been proposed as more appropriate than commercial and individual in-house databases (Mazzeo et al., 2006; Böhme et al., 2012). Initial efforts have already been made in this direction [“FoodBIMS,” (http://bioinformatica.isa.cnr.it/Descr_Bact_Dbase.htm) for 26 species of food borne pathogens (Mazzeo et al., 2006), 200 MALDI-TOF MS spectra for more than 70 bacterial species with links to the freely available web-based application SPECLUST “SpectraBank” (http://www.usc.es/gl/investigacion/grupos/lhica/spectrabank/Database.html; Böhme et al., 2012)]. These open-access databases include results obtained from very few microorganisms and expansion of these databases was not continued. It is imperative that such databases be well curated and be continuously updated. As a first step to achieve this objective the raw data and/or final pooled database files can be submitted as supplementary information along with the publication where the authors reports any expansion or creation of spectral database. Recently, a MALDI-TOF MS database (VibrioBase) was created for fast identification of *Vibrio* spp. that are potentially pathogenic in humans and all main spectra of this newly created database are freely accessible as btmsp-file as supplement with the online article (Erler et al., 2015). It is also expected that MALDI-TOF MS reference spectra created for newly described species will also be made public along with its description as most microbiologist already submit strains with culture collection and sequences with GenBank. Data repositories like Dryad (http://datadryad.org/), which makes data discoverable, freely reusable and citable, can be used as the platform to share newly generated MALDI-TOF MS reference spectra. We hope that more and more researchers will volunteer to make their in-house databases available in public domain.

## Application of MALDI TOF MS in microbial ecology studies

Hundreds of millions of microorganisms populate the earth, each year the number of new validly published names of bacteria increases (Parte, 2014). Microbial ecologist and environmental microbiologist have used simulated culture conditions by mimicking the natural environment to cultivate microorganisms previously considered uncultivable (Kaeberlein et al., 2002). Culturomics is a recently coined term and is defined as “an approach allowing an extensive assessment of the microbial composition by high-throughput culture” (Greub, 2012). Though it is not possible to cultivate the whole microbial communities even after using multiple media and growth condition, but several new microorganisms can be cultivated which are till now reported by cultivation-independent methods. Recently, an isolate L21-Fru-AB belonging to novel phylogenetic lineage within the *Planctomyces*-*Verrucomicrobia*-*Chlamydia* superphylum, was isolated from hypersaline microbial mats using various cultivation techniques including the use of highly selective defined media as well as more universal complex media (Spring et al., 2015). Based on detailed characterization of strain L21-Fru-AB^T^ a novel species and genus *Kiritimatiella glycovorans* is proposed under the novel phylum *Kiritimatiellaeota* comprising environmental sequences allocated previously to the *Verrucomicrobia* subdivision 5 (Spring et al., 2016). The additional advantage of cultivation is that the isolates can be exploited based on their biotechnological potentials. A large number of already known (some unknown as well) microorganisms are generally isolated in bio-prospection studies using, innovative and high-throughput cultivation techniques to culture the dominant, rare, and not-yet-cultivated microbes. Information on the complete or partial characterization of microorganisms play critical role in the selection of potential microbial isolates for further detailed studies. A simple and rapid method of microbial characterization will definitely speed up such research activities. Due to its ability to perform high-throughput identifications and characterization MALDI-TOF MS is the technique of choice in microbial culturomics (Lagier et al., 2012, 2015; Stafsnes et al., 2013; Dubourg et al., 2014). This approach was used to study the microbial diversity of three stool samples, and a total of 340 species of bacteria were identified using MALDI-TOF MS among 32,500 colonies, of which 174 species were recorded for the first time in the human gut (Lagier et al., 2012). In a similar study, 190 bacterial species were identified from 32,000 different colonies isolated from four stool samples, with 9 species described for the first time in the human gut (Dubourg et al., 2014). Application of this technique to identify 2750 isolates of human gut microbiome from 347 individuals (162 with and 185 without diarrhea) in Senegal resulted in 98.8% successful identifications (Samb-Ba et al., 2014). The remaining 1.2% (32 isolates, for which MALDI-TOF MS was not successful) isolates were identified by 16S rRNA gene sequencing, which led to detection of two new species.

The limited number of spectral database, which include very few strains of non-clinical origin, results low identification percentage (43–65%) for microbes isolated from soil, water and other environments (observation in authors' laboratory, data not presented). Similarly, 43% of bacterial isolates from two waste disposal sites from non-ferrous metal industry were not reliably identified using MALDI TOF MS and fewer than 20% were identified at species-level (Kopcakova et al., 2014). Despite these limitations, MALDI TOF MS based characterizations have been done for microorganisms from non-clinical sources including, air samples, sewage sludge, biogas plants, ballast water, coastal caves, marine sponges, spinach-processing plant, plant rhizosphere, plant root nodules, wine, and waste disposal sites from non-ferrous metal industry (Dieckmann et al., 2005; Uhlik et al., 2011; Emami et al., 2012; Hausdorf et al., 2013; Stets et al., 2013; Angelakis et al., 2014; Busquets et al., 2014; Kopcakova et al., 2014; Stantscheff et al., 2014; Usbeck et al., 2014). In a comprehensive analysis 5085 isolates of aerobic, heterotrophic and extremely halophilic bacteria and archea from eight solar salterns distributed among different locations in the Spanish Mediterranean, Canary Islands' Atlantic and Chilean Pacific coasts were retrieved combining large-scale cultivation, and identified using MALDI-TOF MS and 16S rRNA gene analysis (Viver et al., 2015). It has been shown that MALDI-TOF MS is a reliable technique for the identification of diverse microorganisms including both the most commonly encountered aerobic heterotrophic, and the difficult to culture anaerobic, chemotrophic, phototrophic, and extremophilic microorganisms (Krader and Emerson, 2004; Veloo et al., 2011; Biswas and Rolain, 2013; Stantscheff et al., 2014; Vávrová et al., 2014; Shih et al., 2015). MALDI-TOF MS based proteotyping has also been used to eliminate the known species from the unknowns, which resulted into selection of strains of several new species (Dubourg et al., 2014; Samb-Ba et al., 2014; Viver et al., 2015). Tandem study combining MALDI-TOF MS and 16S rRNA gene sequencing of a total of 4243 isolates resulted in to 41 different operational phylogenetic units, of which 22 were regarded as putative new species according to their identity with the closest related type strain sequences (Viver et al., 2015).

In a database-independent approach, MALDI-TOF MS based proteotyping was also be used to recognize identical isolates at specific taxonomic level by clustering them together and selecting representatives of each cluster in order to reduce the number of isolates for subsequent characterization (Dieckmann et al., 2005; Ghyselinck et al., 2011; Stafsnes et al., 2013). De-replication process by this method proved to have higher reproducibility than rep-PCR and considered to be more promising with respect to high-throughput analyses, automation, time, and cost efficiency (Ghyselinck et al., 2011). Stafsnes et al. (2013) also used MALDI-TOF MS as a pre-screening technique for efficient screening of novel pigment producers in a marine bacteria culture collection. A total of 374 isolates from hypersaline sediments of a solar saltern were classified into 25 phenotypic clusters at 52% similarity based on their whole-cell MALDI-TOF MS spectra profile (Munoz et al., 2011). The clustering of same species in a MALDI-TOF MS cluster was confirmed by DDH values above the threshold (70%) of how a species can be circumscribed and intra-cluster diversity results also correspond to the Random Amplified Polymorphic DNA analysis confirming the accuracy of MALDI-TOF MS clustering (Munoz et al., 2011). A total of 244 bacterial colonies isolated from air samples from Makkah, Saudi Arabia were tested by MALDI-TOF MS resulted into identification of 202 isolates and the remaining 42 unidentified isolates were further clustered into 10 clusters and 16S rRNA gene sequencing was performed only for one representative strain per cluster (Angelakis et al., 2014).

Since MALDI-TOF MS based characterization is rapid, easy to operate and relatively inexpensive, it may play a bigger role in quality control and validation of microbial strains preserved in large culture collections, institutes and industries. Hence, it is anticipated that in the upcoming era of microbial culturomics MALDI TOF MS based biotyping will play a key role in bringing a revolution in microbial ecology and diversity studies as it did in the field of clinical diagnosis.

## Conclusions

MALDI-TOF MS is a powerful, cost effective, rapid, and robust technique for identification of microorganisms (bacteria including anaerobes, fungi including yeast, and archaea). Results obtained by this technique are comparable to, if not better than, those obtained by other time consuming and expensive methods of microbial identification. Inadequate and limited size of database and lack of efficient software capable of enhancing the resolution to differentiate closely related species are some of the current challenges of this technology. Continuous expansion of spectral database with representatives from different groups and/or development of well curated open-access mass spectra database is the need of the hour for wide application of the MALDI-TOF MS based identification in environmental microbiology and ecological studies. Use of common protocols and strategies for development of in-house spectra database will improve the identification efficiency of this technique for microbes. High-throughput microbial identification offered by MALDI-TOF MS has a very important role to play in the era of high-throughput cultivation of microbes. It is expected that, with availability of updated, error free and robust database(s) along with optimized methods and protocols, this technique will be able to find its extensive use and will prove to be a valuable asset in the field of environmental microbiology, microbial ecology, and taxonomy.

## Author contributions

PR developed the concept and drafted the review article. OP and YS revised it critically for important intellectual content.

### Conflict of interest statement

The authors declare that the research was conducted in the absence of any commercial or financial relationships that could be construed as a potential conflict of interest.
